# The Influence of the COVID-19 Pandemic on Baseline Concussion Symptom Assessments Among Adolescents

**DOI:** 10.7759/cureus.56235

**Published:** 2024-03-15

**Authors:** Daniella K Rivera, Jeremy Brown, R. Curtis Bay, Tamara C Valovich McLeod

**Affiliations:** 1 Osteopathic Medicine, A. T. Still University School of Osteopathic Medicine, Mesa, USA; 2 Medicine, Kaweah Health Medical Center, Visalia, USA; 3 Health Sciences, A. T. Still University School of Health Sciences, Mesa, USA

**Keywords:** isolation and quarantine, concussion applications, youth sports, quality of life (qol), coronavirus disease 2019 (covid-19)

## Abstract

Introduction

The COVID-19 pandemic resulted in the cancellation of high school sports in spring 2020, a modified resumption of sports in the 2020-2021 academic year, and a return to pre-pandemic sports in 2021-2022. This cancellation had a major impact on the quality of life of adolescent athletes, but it is unknown exactly how these pandemic-driven sports disruptions on athlete baseline (preseason) symptoms affected quality of life. Therefore, the current study retrospectively evaluated symptom inventories from Immediate Post-Concussion Assessment and Cognitive Testing (ImPACT) assessments to determine whether the cancellation of sports during the COVID-19 pandemic affected baseline (preseason) self-reported symptoms among adolescent athletes.

Methods

Our study used a retrospective cohort design to evaluate high school athletes with complete ImPACT assessments in the academic years before (2018-2019 and 2019-2020), during (2020-2021), and after (2021-2022) the pandemic. Specifically, data from a 22-item symptom report called the Post-Concussion Symptom Scale (PCSS) assessed during ImPACT was collected and analyzed using generalized linear models with a Tweedie exponential dispersion model and post hoc Tukey’s honestly significant difference tests. The main outcomes were the total symptom severity score and the affective cluster score. Secondary outcomes were the analysis of the vestibular-somatic, cognitive-sensory, and sleep-arousal symptom clusters.

Results

Of the 104,274 ImPACT assessments, the total symptom severity score on the PCSS was different across years (p<0.001). There were lower symptom scores in 2020-2021 (5.33, 95% CI = 5.13-5.54) than in 2018-2019 (6.82, 95% CI = 6.63-7.01), 2019-2020 (6.94, 95% CI = 6.75-7.14), and 2021-2022 (6.44, 95% CI = 6.25-6.64). The cluster scores on the PCSS for affective, cognitive-sensory, sleep-arousal, and vestibular-somatic were also lower (p<0.001) in 2020-2021 than in 2018-2019, 2019-2020, and 2021-2022.

Conclusion

Contrary to our expectations, total symptom severity and cluster scores on the PCSS during the pandemic (2020-2021) were significantly lower than during the years before and after the pandemic-driven sports disruptions, suggesting the pandemic did not negatively affect these athletes as expected. These results also suggested that self-reported symptoms utilized in the PCSS component of ImPACT may not be as sensitive to sports disruption among adolescent athletes as other quality-of-life measures, especially during the COVID-19 pandemic.

## Introduction

Adolescent involvement in organized sports has been shown to have positive effects on depression, anxiety, and stress management [1,2] and to inculcate lifelong habits of physical activity and fitness [3]. Similarly, the cessation or cancellation of extracurricular activities for adolescents has been shown to have negative effects on various social biomarkers (stress, fatigue, and depression), especially overall mental health [4]. In one study, the elimination of scheduled, organized physical activity in middle and high schools had myriad negative effects, such as increased levels of moderate-to-severe depression and decreased levels of physical activity [5]. At the beginning of the COVID-19 pandemic, there were mass cancellations [6] of in-person activities, including after-school sports activities for youth. Given the benefits of sports participation, the potential repercussions of these pandemic-driven cancellations on adolescents should be investigated.

Quality-of-life (QoL) measures are one way to assess an individual’s overall health. Specifically, QoL provides information about the mental, physical, and social well-being of an individual [7], and the COVID-19 pandemic had a direct influence on various aspects of adolescent QoL [8]. In the United States, the federal government mandated school closures and lockdowns to mitigate the spread of the virus, which caused the cancellation of high school sports [6,9]. Studies [5,10] have shown that this shutdown had negative effects on physical activity, mental health, and overall QoL. Unfortunately, there is little information about the influence of sports disruptions on baseline (preseason) symptoms. Since sports participation has been shown to improve self-esteem and life satisfaction [1], such information, especially regarding affective symptoms that influence QoL, is critical for ongoing sports participation.

Millions of American youths [11] participate in team sports, and the potential for a serious injury, like a concussion, has become commonplace for these athletes [12]. With the increasing prevalence of concussions in youth sports, especially in athletes aged 15 to 19 years [12], evaluations of the effect of these injuries have become a major part of the healthcare provider’s role on and off the field and court. To track the progression of a concussion, student-athletes complete the Immediate Post-Concussion Assessment and Cognitive Testing (ImPACT) tool, which includes the Post-Concussion Symptom Scale (PCSS). The PCSS is a subjective self-report scale used to establish a preseason symptom baseline to gauge the athlete’s ability to safely return to physical activity after a concussive event. The advantage of using ImPACT is that it collects data before starting a sports program and after an injury, providing a before and after clinical picture. ImPACT is not a diagnostic test and should not be used in isolation for managing concussions. Instead, they should be used as integrated tests that objectively track an athlete’s recovery from injury [13,14].

Given the importance of adolescent sport participation on QoL and overall health, the purpose of the current study was to retrospectively evaluate symptom inventories from ImPACT assessments to determine whether the cancellation of sports during the COVID-19 pandemic affected baseline (preseason) self-reported symptoms among adolescent athletes. We hypothesized that adolescents would be negatively affected by the absence of sports during the pandemic, as evidenced by more self-reported symptoms of increased sadness, irritability, nervousness, and emotional distress.

## Materials and methods

The current study used a retrospective cohort design to evaluate high school athletes with complete preseason baseline ImPACT neurocognitive assessments in the academic years before (2018-2019 and 2019-2020), during (2020-2021), and after (2021-2022) the pandemic. Specifically, we used scores from the PCSS part of the ImPACT tool to determine whether pandemic-driven sports disruptions affected their QoL. Data was extracted from an existing database of ImPACT within the state of Arizona. We included these tests in the current study because ImPACT assessments are administered throughout Arizona as part of the Barrow Concussion Network, which is a collaboration between A.T. Still University (ATSU), Barrow Neurological Institute, and the Arizona Interscholastic Association [15]. The current study was considered exempt per the A.T. Still University-Arizona Institutional Review Board.

The ImPACT tool is a computerized neurocognitive concussion tool that evaluates cognitive function and the signs and symptoms of concussion. ImPACT is divided into sections gathering demographic information, the PCSS symptom scale, and computerized testing that measures verbal and visual memory, processing speed, and reaction time [13]. Athletic trainers at each school administer the assessment to all athletes at baseline (preseason) and again after a potential concussive injury.

In the current study, de-identified data from high school athlete participants in the years of interest for this investigation was extracted from the larger ImPACT database by one of the co-authors, who serves as an administrator for the Barrow Concussion Network. Only complete participant data from the four academic years (2018-2019, 2019-2020, 2020-2021, and 2021-2022) was analyzed. To be included in the study, each participant had to complete baseline assessments. For an assessment to be considered complete, it had to be documented as valid according to ImPACT’s internal validity indicator.

The PCSS is a 22-item symptom report on a Likert-type scale, with self-reported scores ranging from 0 or no difficulty to 6 with severe difficulty [16]. The PCSS evaluates four symptom clusters: affective (sadness, irritability, nervousness, and feeling more emotional), cognitive-sensory (fleeing slowed down, feeling mentally “foggy,” difficulty concentrating, difficulty remembering, sensitivity to light, and sensitivity to noise), sleep-arousal (fatigue, trouble falling asleep, sleeping more than usual, sleeping less than usual, and drowsiness), and vestibular-somatic (headache, nausea, vomiting, balance problems, visual problems, dizziness, and numbness) [16].

Collected data were summarized using mean, standard deviation, 95% confidence interval (CI), frequency, and percentages. Comparative analyses of PCSS scores by year were performed using generalized linear models with a Tweedie exponential dispersion link and post hoc Tukey’s honestly significant difference tests. SPSS Statistics version 28 (IBM Corp. Released 2021. IBM SPSS Statistics for Windows, Version 28.0. Armonk, NY: IBM Corp.) was used for all analyses. An alpha of 0.05 (two-tailed) was used as the criterion for statistical significance.

## Results

The current study analyzed 104,274 PCSS scores from ImPACT assessments for the four academic years of 2018-2019, 2019-2020, 2020-2021, and 2021-2022 (Table 1). Across all four academic years, there were 41,801 (40.09%) female participants compared to 62,473 (59.91%) male participants. This consisted of 58,724 (56.32%) high school and 1,933 (1.85%) junior high student-athletes, as well as 43,617 (41.83%) student-athletes who elected to not identify and “skip” their level of education on the assessment. The majority of ImPACT assessments came from student-athletes who elected to “skip” their sports selection (n = 60,065 (57.6%)), followed by football (n = 17,638 (16.92%)).

**Table 1:** Completed ImPACT assessments by academic year ImPACT: Immediate Post-Concussion Assessment and Cognitive Testing

Academic Year	No. Completed Assessments
2018-2019	30,875 (29.61%)
2019-2020	31,273 (29.99%)
2020-2021	16,702 (16.02%)
2021-2022	25,424 (24.38%)
Total	104,274 (100%)

**Figure 1:**
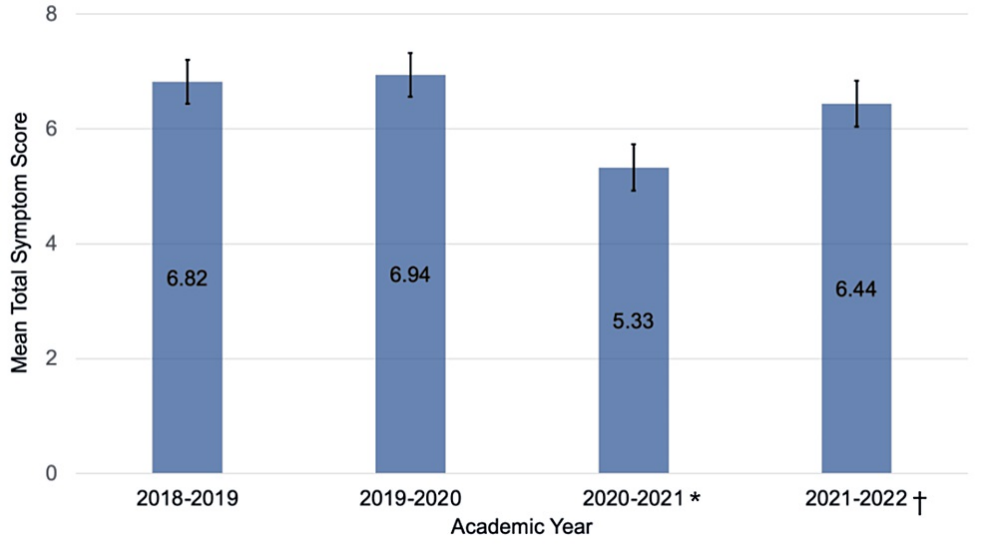
Total symptom severity scores on the PCSS by academic year Error bars represent 95% confidence intervals. *Scores were significantly lower than during all other years (p<0.001, Tukey’s honestly significant difference). †Scores were significantly lower than during 2018-2019 and 2019-2020 (p<0.001). PCSS: Post-Concussion Symptom Scale

The total symptom severity score on the PCSS was different between years (p<0.001) (Figure 1). There were lower total symptom scores in 2020-2021 during the pandemic (5.33, 95% CI = 5.13-5.54) than in 2018-2019 (6.82, 95% CI = 6.63-7.01), 2019-2020 (6.94, 95% CI = 6.75-7.14), and 2021-2022 (6.44, 95% CI = 6.25-6.64). The 2021-2022 academic year also had lower total symptom scores than the 2018-2019 and 2019-2020 academic years (p<0.001). No differences in total symptom scores were found between the 2018-2019 and 2019-2020 academic years (p=0.376).

Similarly, scores for the four symptom clusters-affective, cognitive-sensory, sleep-arousal, and vestibular-somatic-of the PCSS were lower in 2020-2021 during the pandemic (all p<0.001) than in 2018-2019, 2019-2020, and 2021-2022 (Table 2). No other differences were found between academic years for the affective cluster and the cognitive-sensory cluster (all p>0.05). The 2021-2022 academic year also had lower scores for the vestibular-somatic and sleep-arousal clusters than the 2018-2019 and 2019-2020 academic years (all p<0.001). No other differences were found between academic years for the vestibular-somatic and sleep-arousal clusters (all p>0.05).

**Table 2:** Symptom profile cluster scores of the PCSS by academic year *Scores for all four symptom profile clusters were significantly lower during 2020-2021 than during other academic years (p<0.001). †Scores for the vestibular-somatic and sleep-arousal symptom profile clusters were significantly lower during 2021-2022 than during 2018-2019 and 2019-2020 (p<0.001). PCSS: Post-Concussion Symptom Scale

Symptom Profile Cluster	Mean (95% CI) Scores by Academic Year			
	2018-2019	2019-2020	2020-2021*	2021-2022†
Affective	1.78 (1.70-1.86)	1.79 (1.71-1.86)	1.47 (1.39-1.56)	1.72 (1.65-1.81)
Cognitive-sensory	1.83 (1.76-1.91)	1.90 (1.82-1.98)	1.46 (1.37-1.55)	1.83 (1.75-1.92)
Sleep-arousal	1.98 (1.91-2.05)	2.03 (1.95-2.10)	1.57 (1.49-1.66)	1.84 (1.76-1.91)
Vestibular-somatic	0.85 (0.80-0.90)	0.86 (0.81-0.91)	0.53 (0.49-0.58)	0.71 (0.66-0.75)

## Discussion

In the current study, we retrospectively evaluated symptom inventories from ImPACT assessments to determine whether the cancellation of sports during the COVID-19 pandemic affected baseline (preseason) self-reported symptoms among adolescent athletes. We found statistically significant differences in total symptom severity and cluster scores on the PCSS between academic years before, during, and after the pandemic. Contrary to our hypothesis that student-athletes would be more likely to experience social isolation and emotional distress because of sports cancellations, which would lead to declining QoL [17], these adolescents experienced significantly lower total symptom score severity and lower symptom cluster scores during the 2020-2021 academic year than during the years before and after the pandemic.

Our study findings contradict those of McGuine et al. [5]. In that study, depression and QoL of adolescent athletes were evaluated using the Patient Health Questionnaire-9 (PHQ-9) and the Pediatric Quality of Life Inventory 4.0 (PedsQL), respectively [5]. The authors compared outcomes before and during the pandemic and reported increased depression and decreased QoL during the pandemic [5]. In our study, the affective cluster was significantly lower during the pandemic than before or after, suggesting these adolescent athletes had better overall QoL. These contrary findings may be due to differences in the assessment tools. The PHQ-9 and the PedsQL are more comprehensive exams than the PCSS and address a more diverse population. For example, the PHQ-9 assesses somatic, anxiety, and depressive symptoms [18], and the PedsQL evaluates physical, emotional, social, and school functioning [19].

Other studies [5,17,20-22] have also reported that the pandemic had a significant negative effect on adolescent mental health and QoL. An Australian study from Elliot et al. [17] found that the lost sporting season for youths brought on an “emotional struggle” and “provided a sense of disappointment and 'mourning.’” This is consistent with a European study from Panchal et al. [21], with children and adolescents commonly reporting symptoms of anxiety and depression. There may be several reasons student-athletes in our study had better self-reported QoL outcomes during the 2020-2021 academic year than in academic years before and after the pandemic. Perhaps these students considered the virtual schooling, sports cancellations, business closures, and social isolation required by the pandemic as normal. Further, during the first wave of lockdowns and school closures, students may have been relieved to stay home from school for extended periods. Perhaps the usual mental and QoL stressors experienced by adolescents, such as bullying, excessive amounts of homework, or burnout from academics or sports practice, were alleviated by the adoption of virtual schooling because of school closures [23]. However, a study by Cingel et al. [24] suggested students who attended school virtually during the pandemic had higher rates of depression, lower satisfaction with peer connection, and lower levels of school satisfaction than students who attended school in person [24].

Another difference between our results and those of other studies [5,20-21] may be related to when or where the study was conducted. For instance, previous studies [5,20-21] recruited participants between early 2020 and spring 2020; this period included the start of school closures and sports cancellations. Conversely, the majority of students in our study completed their ImPACT assessments in the fall of 2020 (August-September). This only included student-athletes in Arizona, compared to out-of-state [5] or international studies [20-21]. Given that sports participation was resumed in Arizona in the fall of 2020, with some modifications for the pandemic [25], this difference in results because of the timing of data collection is reasonable. For example, despite having to wear masks and adhering to other restrictions [25], student-athletes who completed ImPACT assessments in the fall of 2020 knew they would be playing, which may have influenced their symptom scores. Therefore, our statistically significant lower scores during the 2020-2021 season for all analyzed outcomes may be explained by the resumption of sports at the beginning of that academic year.

Several limitations should be considered when interpreting the results of the current study. Although Riegler et al. [26] successfully used PCSS scores from ImPACT assessments as a validated screen for depression in their study, a limitation of our study may be that the symptom profile scores of the PCSS are not as sensitive for measuring overall QoL as they were for depression. For example, of the 22-item self-reported symptoms on the PCSS, only five assess anxiety and mood [16]. However, this study [26] assessed collegiate athletes compared to our adolescent population. Another limitation of the current study may be related to how our student-athletes answered items on the PCSS. For instance, instead of reporting possible symptoms they were currently experiencing or in relation to the pandemic shutdown, they may have answered items based on how they perceived they should score or how their symptoms would be affected by a concussion. Other study limitations are related to our population. Although we had a large sample size, only adolescent athletes in Arizona were included, and the completed number of assessments during the pandemic was almost half that of previous years. Because we did not include student-athletes nationwide or assess ImPACT scores from other states, the generalizability and applicability of our results are limited and may not correspond to the general adolescent athlete population. Further, we did not exclude participants with pre-existing mental health diagnoses, learning disabilities, or previous concussions, which may have affected their PCSS scores. However, we included these participants for all academic years to preserve consistency. Another limitation is related to the administration of ImPACT assessments. Although ImPACT is a standardized test, our study was retrospective, so we were unable to control the mode of delivery. Further, the test-taking environment may differ from high school to high school, which could affect scores. Similarly, we could not control for pandemic-related cancellations or sports modifications. Although the Arizona Interscholastic Association, a regulatory body for high school athletics in Arizona, followed the Centers for Disease Control and Prevention guidelines [27] for sports resumption, it is unknown whether other states did so as well. Different communities and states had different timelines for shutdowns at the beginning of the pandemic and resumption of activities later in the pandemic. Therefore, student-athletes from states with different pandemic-related timelines and sports disruptions for resumption of activities may have different ImPACT and PCSS scores than participants in our study.

## Conclusions

In the current retrospective study, we hypothesized that symptom inventories from ImPACT assessments would show that the cancellation of sports during the COVID-19 pandemic affected self-reported symptoms (affective, cognitive-sensory, sleep-arousal, and vestibular-somatic) of adolescent athletes. Additional studies should be conducted to determine whether cancellations affect symptoms in a nationwide population of adolescent athletes. Although various reasons may explain variation, self-reported symptom data from the PCSS, obtained during preseason ImPACT assessments, may not be as sensitive as other QoL measures for evaluating the effects of the COVID-19 pandemic on overall adolescent health.
